# Supplementary material for: Mutations in the acetylation hotspots of Rbl2 are associated with increased risk of breast cancer
Source: PLoS One. 2022 Apr 6;17(4):e0266196. doi: 10.1371/journal.pone.0266196 (PMC8985964; doi:10.1371/journal.pone.0266196)

S1 Table. Primers used for mutation analysis of the pocket domain of Rbl2 gene.

Exon No's	Primers Sequence (3′-5′)		Product Size (bp)
	Forward	Reverse	
19	CTATCACCAAGGGTGTGCTC	GGACAGTTTTGCCAGCTAGA	327
20	GCAGGTAGCTGTTCCCTTTC	ATACGGACCAGGTGAATGGT	391
21	CTGGTTTAGCACACCTCTTCA	GCATCTGAAGCATTTTGAGG	311
22	AAACTCTTCCAGGGTAAACTGC	TCATCAGCTGGATTAAGGAAAA	373

S2 Table. Mutation frequencies in various exons of *Rbl2/p130* gene among breast cancer patients

Exons	Mutations in Blood (Germline)	Mutations in Tissues (Somatic)	Missenses	Silent	Insertion	Deletion	Novel	Reported	Total
Exon-19	08	03	07	01	03	--	10	01	11
Exon-21	28	--	18	10	--	--	24	04	28
Exon-22	15	2	14	03	--	01	14	03	17

S3 Table. Mutations in exon-19 (g.46510-46615) of *Rbl2/p130* gene among breast cancer patients

**Variables**	**Genomic Location**	**Change of Codon**	**Change of Amino Acids**	**Tumor Grades (n = 200)**			**Diseased (n = 200)**	**Controls (n = 200)**	**Odds ratio**	**CI 95%**		***p* value**
				GI	GII	GIII				**lower**	**upper**	
**No. of times exon mutated in blood**				**21**	**27**	**23**	**71**	**7**	**15.18**	**6.77**	**34.04**	**<0.0001**
**No. of times exon mutated in tissues**				**22**	**29**	**23**	**74**	**5**	**22.91**	**9.01**	**58.23**	**<0.0001**
**Germline mutations (Blood)**												
Locations	g.46518 A>G*	AGA to AGG	Arg to Arg	14	11	9	34	0	83.09	5.06	1365.60	0.0020
	g.46520 G>A*	AGT to AAG	Ser to lys	26	33	23	82	11	11.94	6.11	23.34	<0.0001
	g.46521T>G	AGT to AAG	Ser to lys	6	7	5	18	6	3.20	1.24	8.23	0.0160
	g.46522 G>A	GTT to ATG	Val to Meth	11	12	7	30	7	4.87	2.08	11.36	0.0003
	g.46522 G>T	GTT to TGT	Val to Cys	9	6	5	20	0	45.54	2.74	758.48	0.0078
	g.46523 T>G	GTT to TGT	Val to Cys	7	9	6	22	0	50.55	3.04	839.35	0.0062
	g.46524 T>G	GTT to ATG	Val to Meth	8	11	13	32	0	77.34	4.70	1272.66	0.0023
	g.46526 T>C	TTG to TCG	Leu to Ser	3	5	6	14	0	31.18	1.85	526.35	0.0171
**Somatic mutations (Tissues)**												
Locations	g.46520 AGA **	AGA	Arg	2	3	7	12	0	26.59	1.56	452.29	0.0233
	g.46520 TAG **	TAG	Stop	2	4	6	12	0	26.59	1.56	452.29	0.0233
	g.46528 TTCC**	ATA<CAT	Ile to His	3	6	7	16	0	35.86	2.14	602.03	0.0129

(**reported*,***insertion*)

S4 Table. Mutations in exon-21 (g.48295-48459) of *Rbl2/p130* gene among breast cancer patients

Variables	Genomic Location	Change of Codon	Change of Amino Acids	Tumor Grades (n = 200)			Diseased (n = 200)	Controls (n = 200)	Odds ratio	CI 95%		*p* value
				GI	GII	GIII				lower	upper	
**No. of times exon mutated in blood**				**29**	**31**	**34**	**94**	**11**	**15.24**	**7.81**	**29.73**	**<0.0001**
**No. of times exon mutated in tissues**				**32**	**34**	**34**	**100**	**13**	**14.39**	**7.69**	**26.92**	**<0.0001**
**Germline (Blood & Tissues)**												
Locations	g.48296 T>G	ATG to AGG	Meth to Arg	6	9	7	22	0	50.55	3.04	839.35	0.0062
	g.48299 A>G	GAT to GGG	Asp to Gly	11	24	27	62	7	12.39	5.50	27.89	<0.0001
	g.48300 T>G	GAT to GGG	Asp to Gly	2	3	5	10	0	22.10	1.29	379.82	0.0329
	g.48303 T>C	GCT to GCC	Ala to Ala	6	5	7	18	0	40.65	2.43	679.39	0.0099
	g. 48306 T>C	CCT to CCC	Pro to Pro	2	6	6	14	0	31.18	1.85	526.35	0.0171
	g.48309 A>C	CCA to CCC	Pro to Pro	13	14	17	44	3	18.52	5.64	60.78	<0.0001
	g.48315 T>C	TCT to TCC	Ser to Ser	6	5	3	14	0	31.18	1.85	526.35	0.0171
	g.48332 G>A	AGA to AAA	Arg to Lys	16	19	25	60	7	11.82	5.24	26.63	<0.0001
	g.48336 A>G	ACA to ACG	Thre to Thre	5	10	11	26	0	60.90	3.68	1006.69	0.0041
	g.48340 T>C	TCC to CCC	Ser to Pro	13	18	19	50	5	13.00	5.06	33.40	<0.0001
	g.48350 G>A*	CGA to CAA	Arg to Gln	33	27	18	78	6	20.67	8.74	48.89	<0.0001
	g.48375 T>G	CCT to CCG	Pro to Pro	13	11	4	28	0	66.25	4.02	1093.28	0.0034
	g.48382 A>T	ATT to TTT	Ile to Phe	14	19	25	58	10	7.76	3.83	15.71	<0.0001
	g.48385 T>C	TCC to CCC	Ser to Pro	22	19	21	62	7	12.39	5.50	27.89	<0.0001
	g.48399 T>G	AAT to AAG	Asn to Lys	13	34	49	96	12	14.46	7.58	27.60	<0.0001
	g.48407 T>G*	ATG to AGG	Meth to Arg	6	5	3	14	0	31.18	1.85	526.35	0.0171
	g.48409 C>T	CTT to TTT	Leu to Phe	13	11	8	32	0	77.34	4.70	1272.66	0.0023
	g.48414 T>C	TCT to TCC	Ser to Ser	10	15	11	36	0	88.98	5.42	1460.81	0.0017
	g.48417 T>C	CCT to CCC	Pro to Pro	5	4	3	12	0	26.59	1.56	452.29	0.0233
	g.48419 G>A*	CGA to CAA	Arg to Gln	38	33	25	96	3	60.62	18.75	195.98	<0.0001
	g.48421 G>A	GAA to AAA	Glu to Lys	15	13	10	38	0	95.01	5.79	1558.38	0.0014
	g.48426 G>A	AAG to AAA	Lys to Lys	19	18	29	66	6	15.93	6.71	37.80	<0.0001
	g.48427 A>T	ATT to TTT	Ile to Phe	7	6	6	19	0	43.08	2.58	718.71	0.0088
	g.48432 C>T*	TTC to TTT	Phe to Phe*	31	34	39	104	22	8.77	5.20	14.78	<0.0001
	g.48448 A>G	AGT to GGC	Ser to Gly	24	31	39	94	13	12.76	6.81	23.88	<0.0001
	g.48449 G>T	AGT to ATC	Ser to Ile	28	37	41	106	18	11.40	6.52	19.93	<0.0001
	g.48450 T>C	AGT to ATC	Ser to Ile	17	25	44	86	9	16.01	7.76	33.05	<0.0001
	g.48458 A>G	AAG to AGG	Lys to Arg	27	34	41	102	22	8.42	4.99	14.20	<0.0001

(**reported)*

S5 Table. Mutations in exon-22 (g.56754-58273) of *Rbl2/p130* gene among blood samples of breast cancer patients

Variables	Genomic Location	Change of Codon	Change of Amino Acids	Tumor Grades (n = 200)			Diseased (n = 200)	Controls (n = 200)	Odds ratio	CI 95%		*p* value
				GI	GII	GIII				lower	upper	
**No. of times exon mutated in blood**				**29**	**33**	**36**	**98**	**7**	**26.49**	**11.86**	**59.16**	**<0.0001**
**Germline mutations (Blood)**												
Locations	g.56755 G>A	AGA to AAC	Arg to Asn	28	39	45	112	21	10.85	6.38	18.46	<0.0001
	g.56756 A>C	AGA to AAC	Arg to Asn	20	37	41	98	13	13.82	7.38	25.87	<0.0001
	g.56758 T>G	CTG to CGG	Leu to Arg	17	32	45	94	9	18.82	9.13	38.82	<0.0001
	g.56761 G>A	AGA to AAA	Arg to Lys	18	27	41	86	11	12.96	6.64	25.31	<0.0001
	g.56763 G>A	GAA to AAA	Glu to Lys	24	36	42	102	4	51.00	18.24	142.57	<0.0001
	g.56790 G>A	GAA to AAA	Glu to Lys	35	39	34	108	28	7.21	4.43	11.73	<0.0001
	g.56793 A>C*	ACT to CCC	Thr to Pro	47	48	17	112	34	6.21	3.91	9.87	<0.0001
	g.56795 T>C*	ACT to CCC	Thr to Pro	31	34	29	94	0	355.82	21.87	5787.86	<0.0001
	g.56807 G>A*	AAG to AAA	Lys to Lys*	19	37	46	102	18	10.52	6.02	18.39	<0.0001
	g.56810 A>G	AGA to AGG	Arg to Arg	32	35	45	112	14	16.91	9.18	31.14	<0.0001
	g.56814 A>T	ATT to TTT	Ile to Phe	29	31	46	106	16	12.97	7.25	23.20	<0.0001
	g.56828 T>G	GAT to GAG	Asp to Glu	4	9	9	22	0	50.55	3.04	839.35	0.0062
	g.56843 T>C	CCT to CCC	Pro to Pro	10	15	17	42	1	52.89	7.20	388.60	0.0001
	g.56845 C>G	GCA to GGA	Ala to Gly	12	13	13	38	1	46.68	6.34	343.68	0.0002
	g.56851 G>A	AGA to AAA	Arg to Lys	18	29	39	86	3	49.54	15.31	160.26	<0.0001
**No. of times exon mutated in tissues**				**29**	**35**	**36**	**100**	**19**	**9.53**	**5.51**	**16.48**	**<0.0001**
**Somatic mutations (Tissues)**												
Locations	g.56817 C>T*	CTT to TTT	Leu to Phe*	35	28	41	104	26	7.25	4.41	11.92	<0.0001
	g.56840 A del***	TCA to CCC	Ser to Pro***	17	32	39	88	14	10.44	5.67	19.23	<0.0001

(**reported, **insertion, ***deletion*

S6 Table: Average Root mean square deviation (RMSD in Å) and radius of gyration (R_g in Å) values for Cyc-D1/CDK4–Rbl2 wild-type and mutated complexes.

Complexes	RMSD in Å	Radius of gyration in Å
Cyc-D1–CDK4–Rbl2 (Wild-Type)	2.02 ± 0.39	27.34 ± 0.13
Cyc-D1–CDK4–Rbl2 (K1083R)	2.24 ± 0.38	28.25 ± 0.12
Cyc-D1–CDK4–Rbl2 (K1083Q)	1.63 ± 0.31	27.10 ± 0.10
Cyc-D1–CDK4–Rbl2 (K1083D)	1.94 ± 0.30	28.23 ± 0.18

S7 Table: Estimated MM-PBSA binding energy (in kcal/mol) for Cyc-D1/CDK4 complex with Rbl2 protein, as calculated from MD trajectory. After discarding the first 4 ns of initial trajectory, a total of 400 frames (snapshot of complexes) were collected after a regular intervals of 10 frames spanning over the remaining 16 ns of corresponding trajectory. The individual contribution form van der Waal's (VDW), electrostatics (EEL), polar solvation free energy (EPB) and non-polar solvation free energy (ENPOLAR) is also given.

**Complexes**	**Energy Contribution in kcal/mol**				
	**Total Binding Energy ± Standard Deviation (Standard Error of Mean)**	**VDW**	**EEL**	**EPB**	**ENPOLAR**
Cyc-D1/CDK4 – Rbl2 (Wild-Type)	-134.26 ± 15.57 (0.78)	-179.93	-1041.61	1109.49	-22.21
Cyc-D1/CDK4 – Rbl2 (K1083R)	-128.60 ± 13.4 (0.67)	-153.24	-961.46	1003.60	-17.49
Cyc-D1/CDK4 – Rbl2 (K1083Q)	-131.97 ± 15.91 (0.79)	-229.85	-1115.77	1240.81	-27.16
Cyc-D1/CDK4 – Rbl2 (K1083D)	-109.75 ± 18.22 (0.91)	-149.13	-822.96	879.01	-16.67

S1 Fig: (a) Overlapped lowest energy models of docked Cyc-D1/CDK4 complex with Rbl2 proteins (wild-type and mutated) as generated from GRAMM-X protein-protein docking web server v.1.2.0. The Cyc-D1/CDK4 complex are represented cyan/red, while the Rbl2 is as purple. (b; wild-type, K1083), orange (c; K1083R), green (d; K1083Q) and yellow (e; K1083D). The amino acid residue 1083 is displayed in VDW representation.

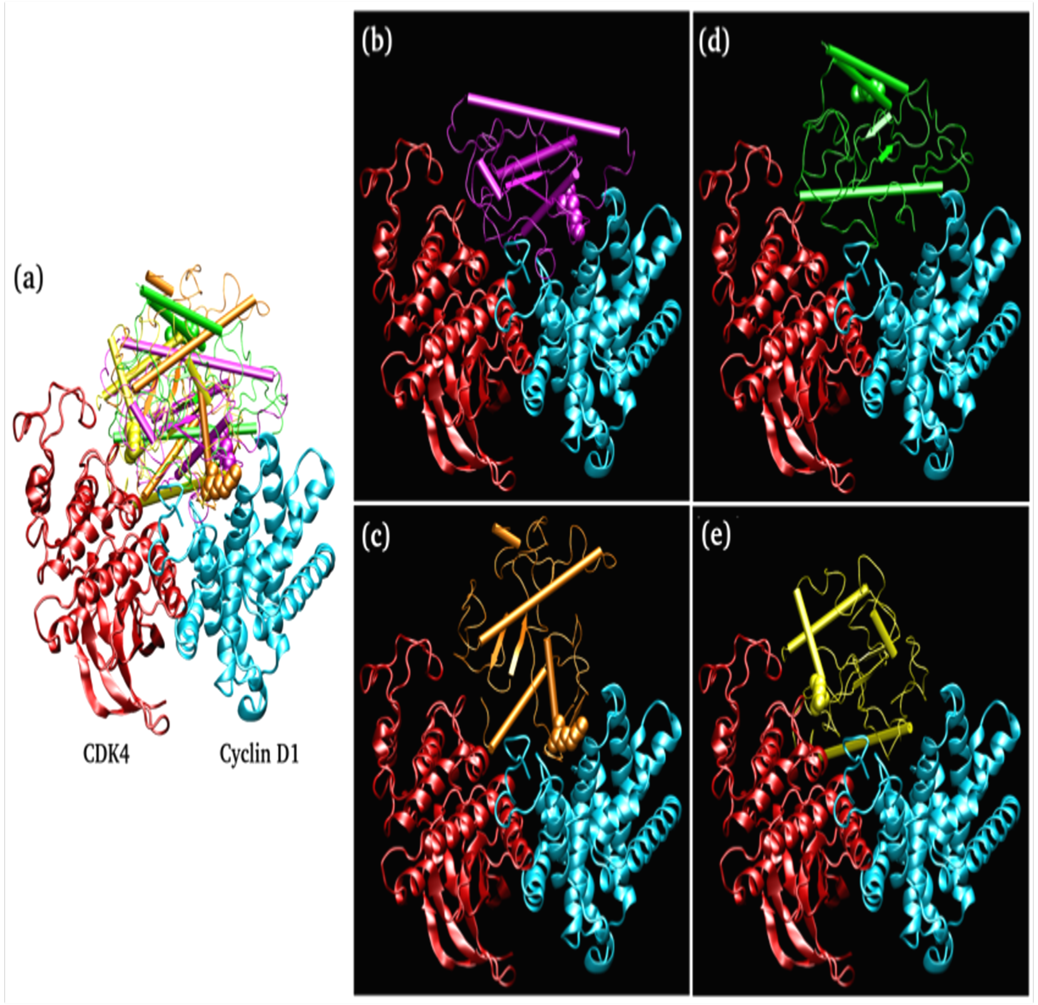

Supplement: S1 File — (PDF) [file pone.0266196.s001.pdf]
