# Supplementary material for: Mutations in the acetylation hotspots of Rbl2 are associated with increased risk of breast cancer
Source: PLoS One. 2022 Apr 6;17(4):e0266196. doi: 10.1371/journal.pone.0266196 (PMC8985964; doi:10.1371/journal.pone.0266196)
Supplement: S2 File — (PDF) [file pone.0266196.s002.pdf]

Exon 19

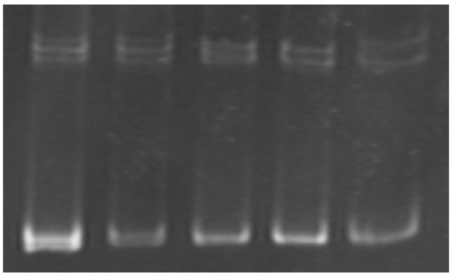

Exon 19.2

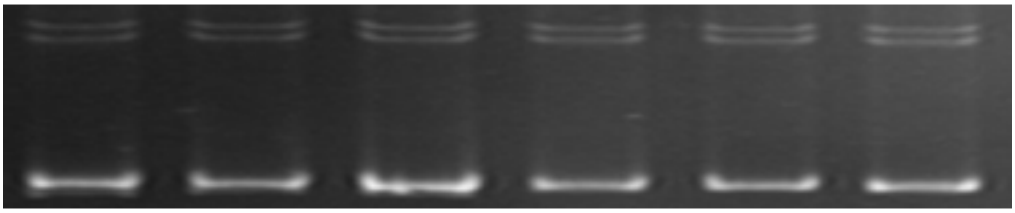

Exon 21.1

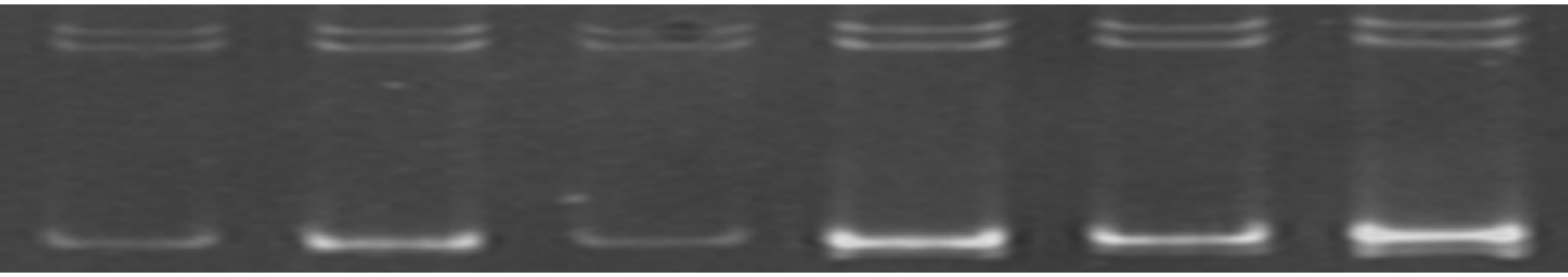

Exon 21.2

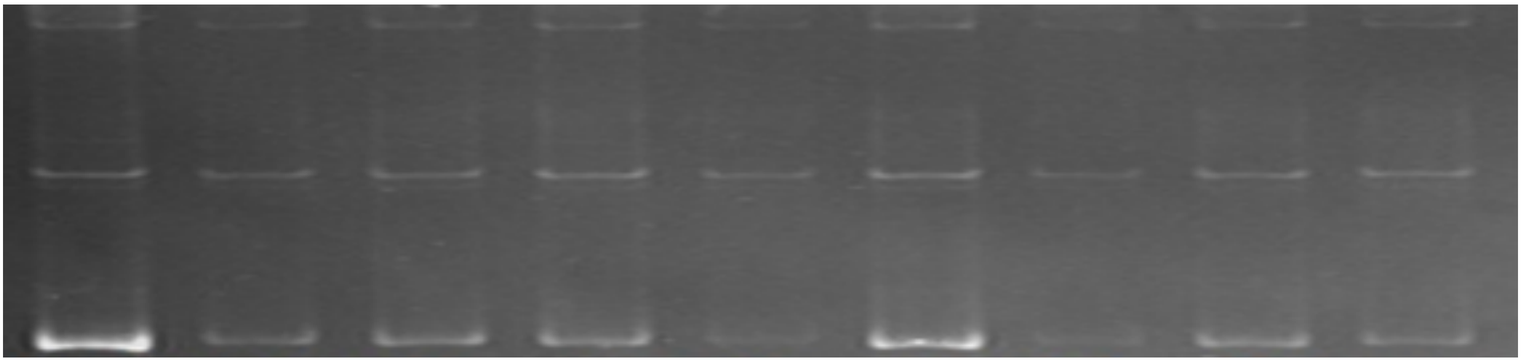

Exon 21.3

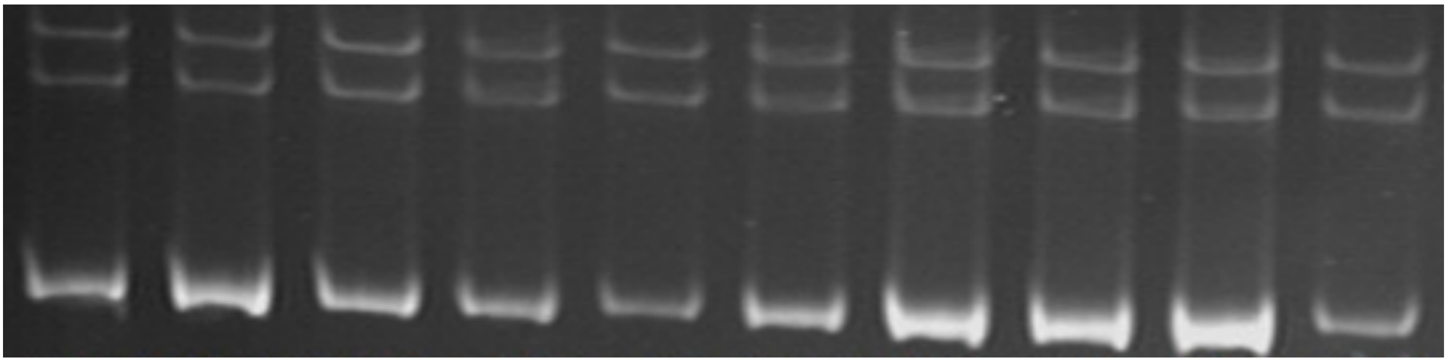

Exon 21.4

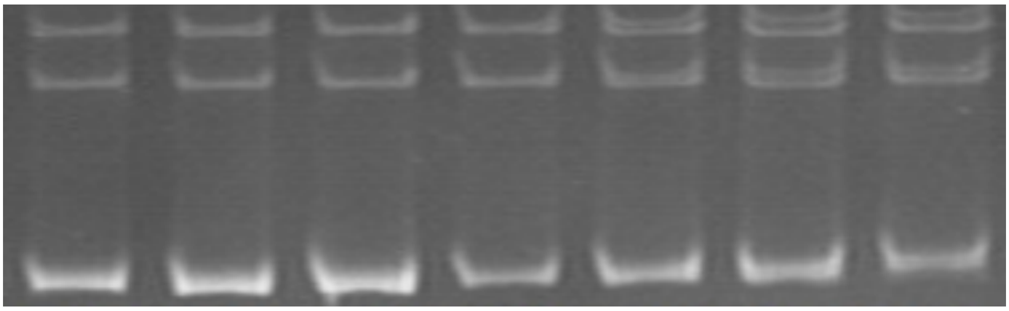

Exon 21.5

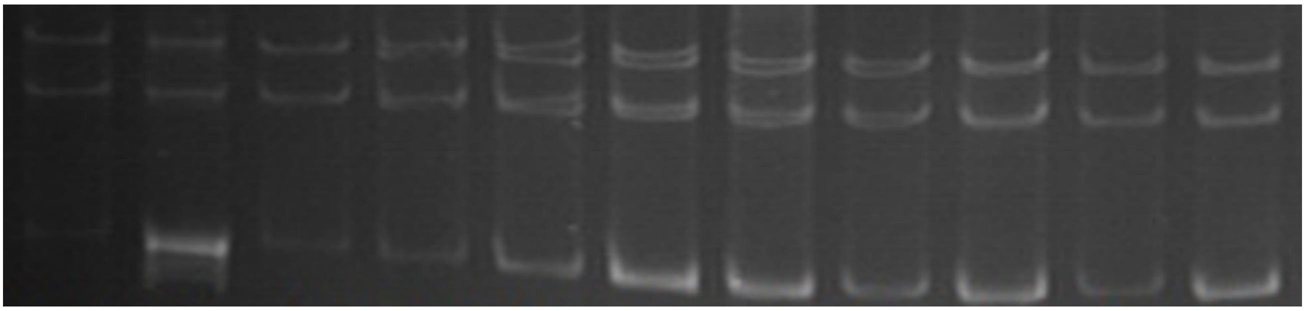

Exon 21.6

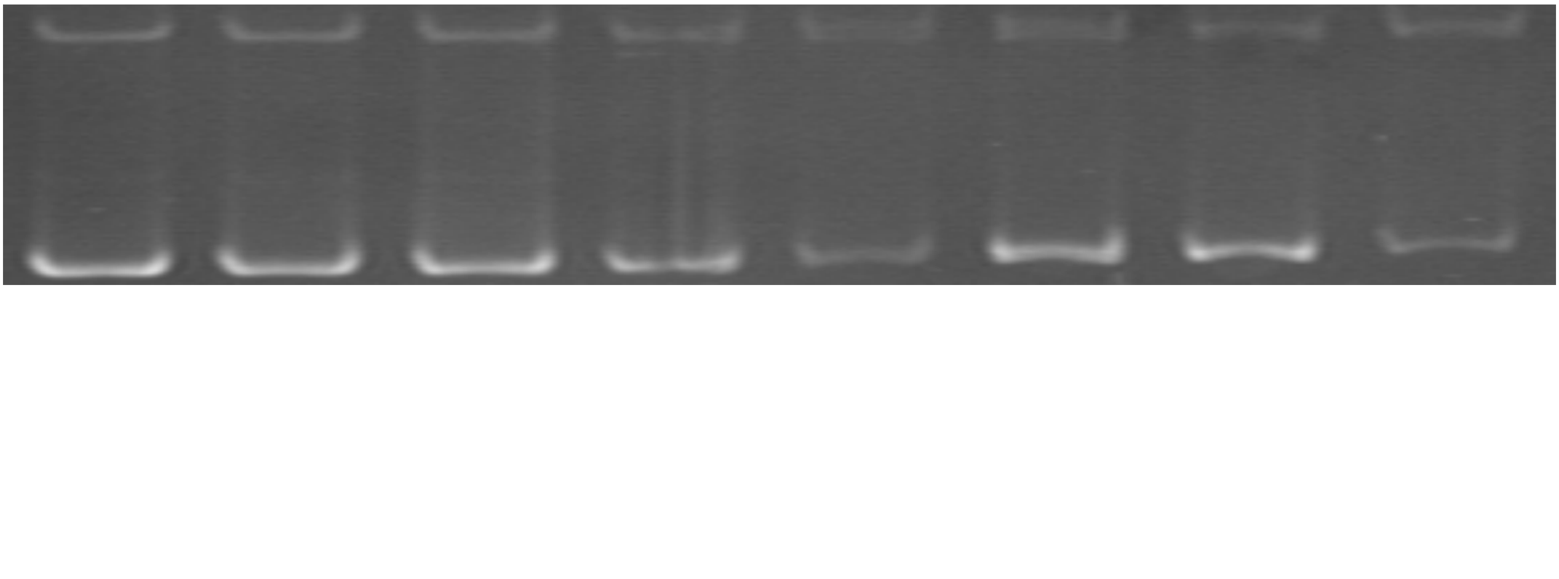

Exon 21.7

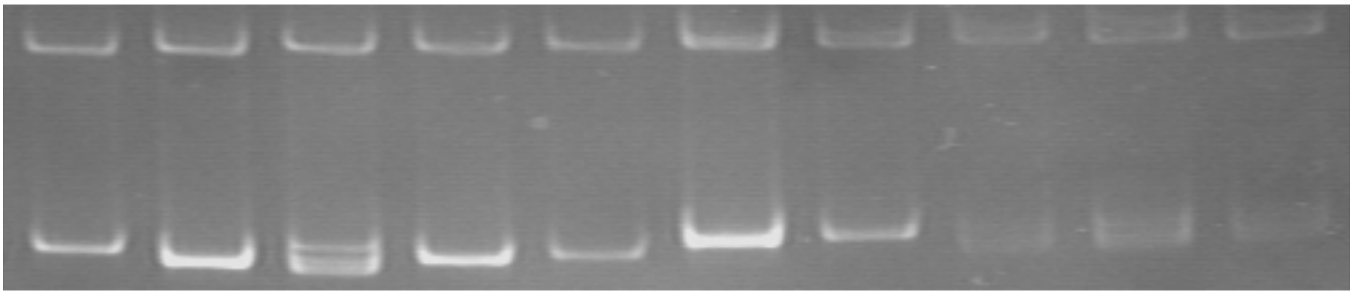

Exon 21.8

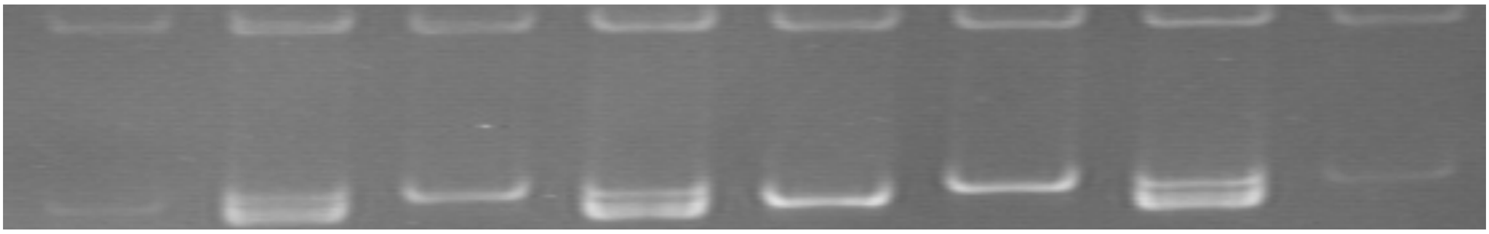

Exon 21.9

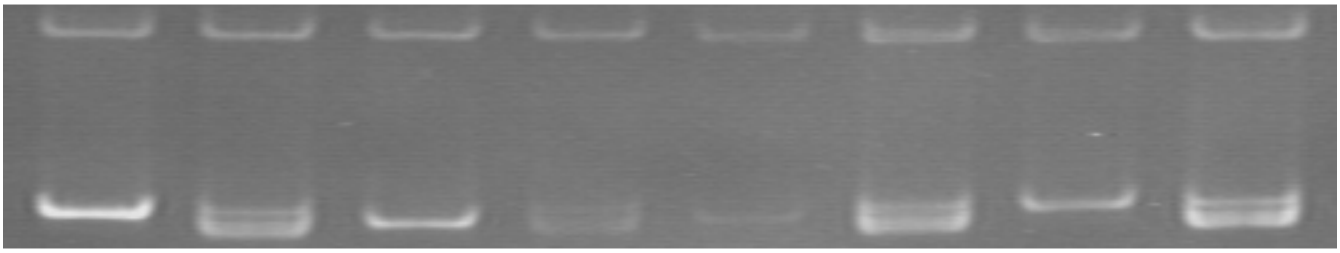

Exon 21.10

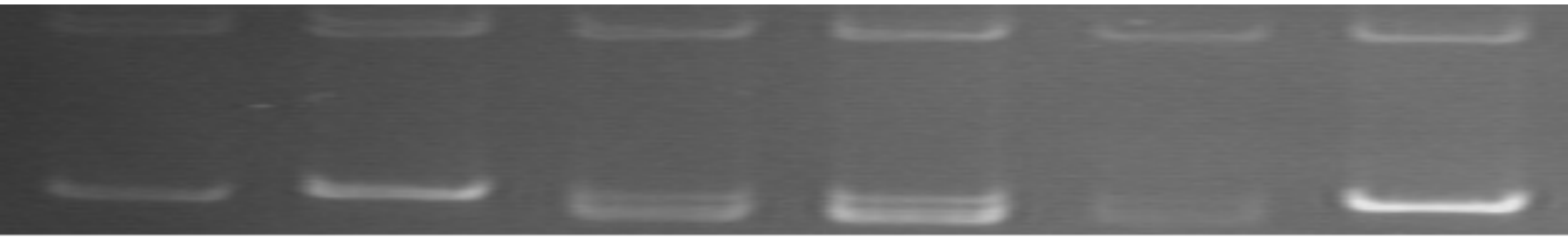

Exon 21.11

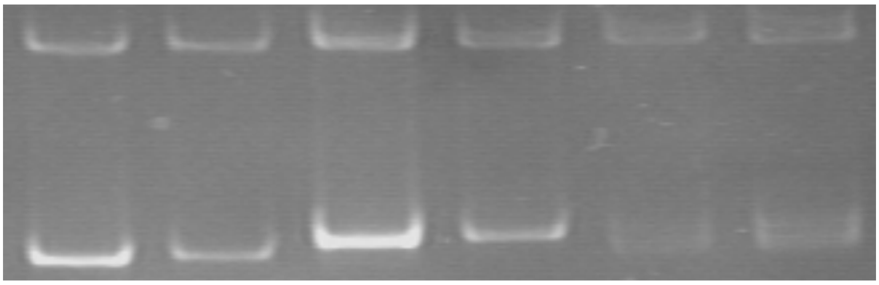

Exon 21.12

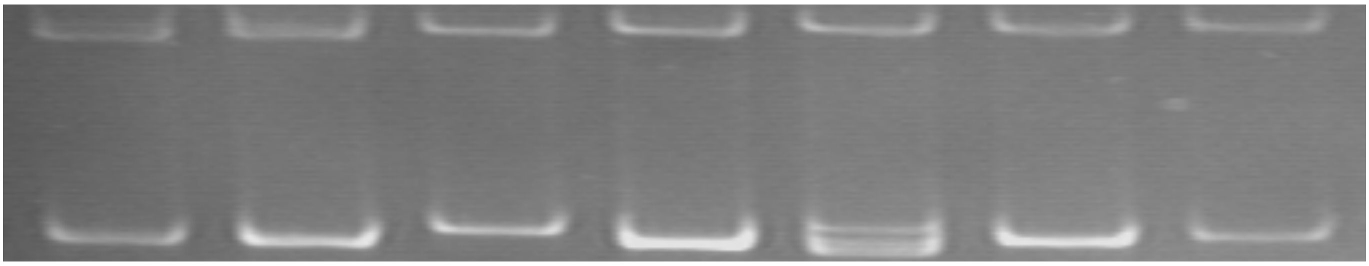

Exon 21.13

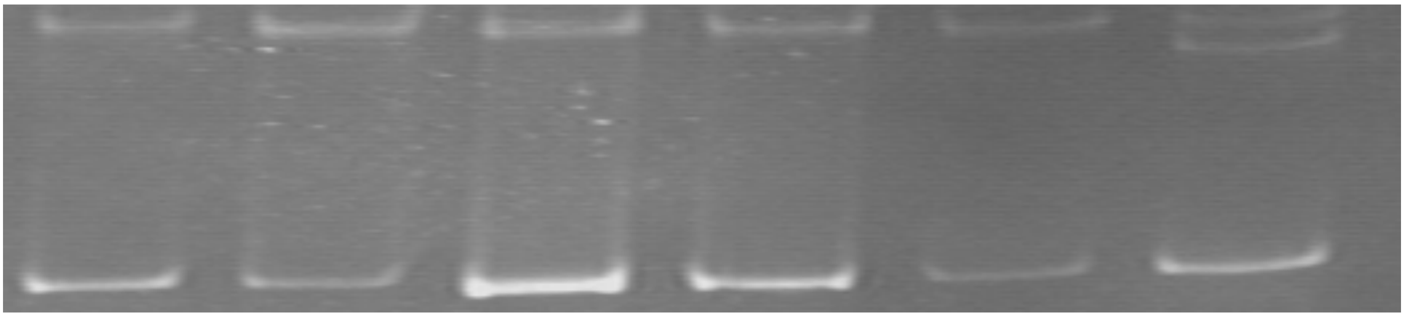

Exon 21.14

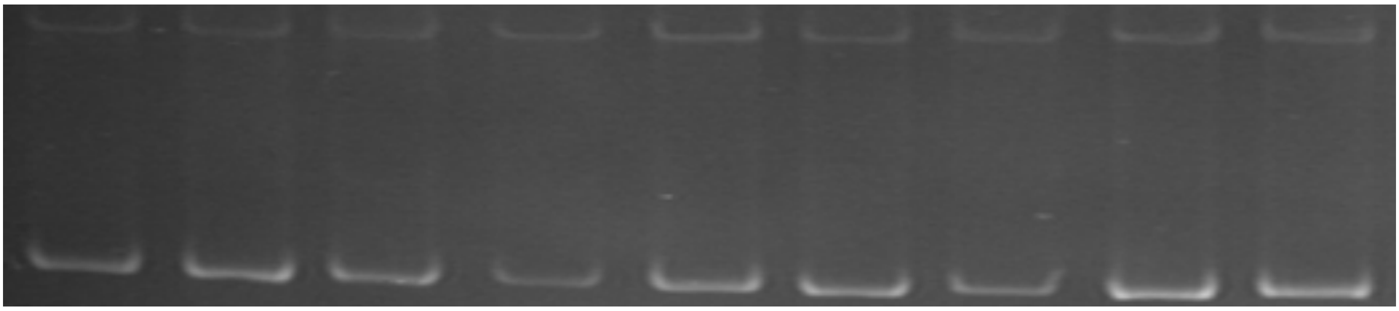

Exon 21.15

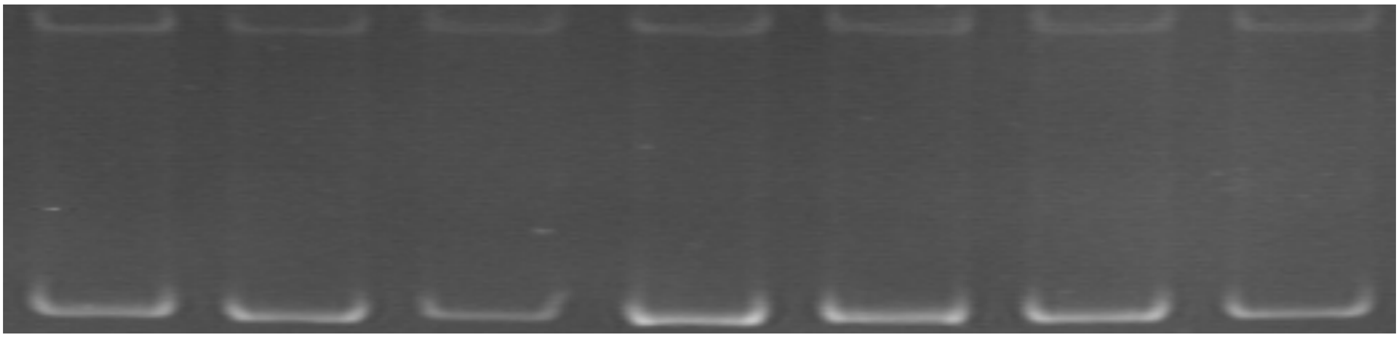

Exon 21.16

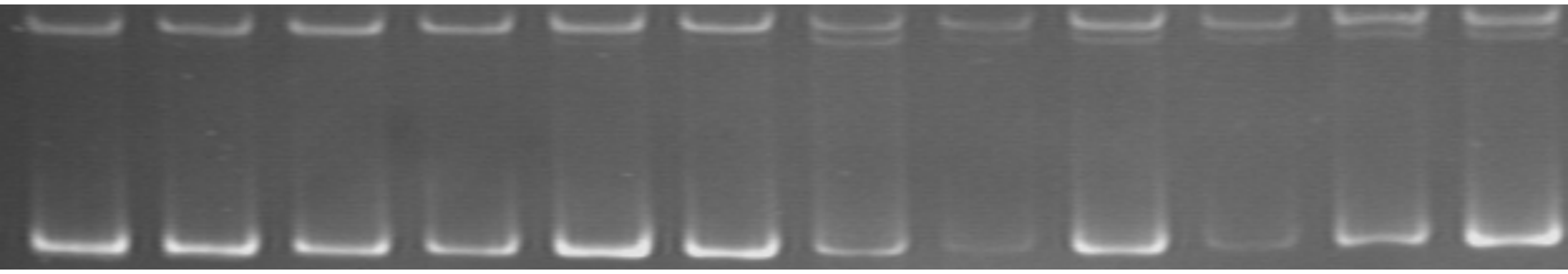

Exon 21.17

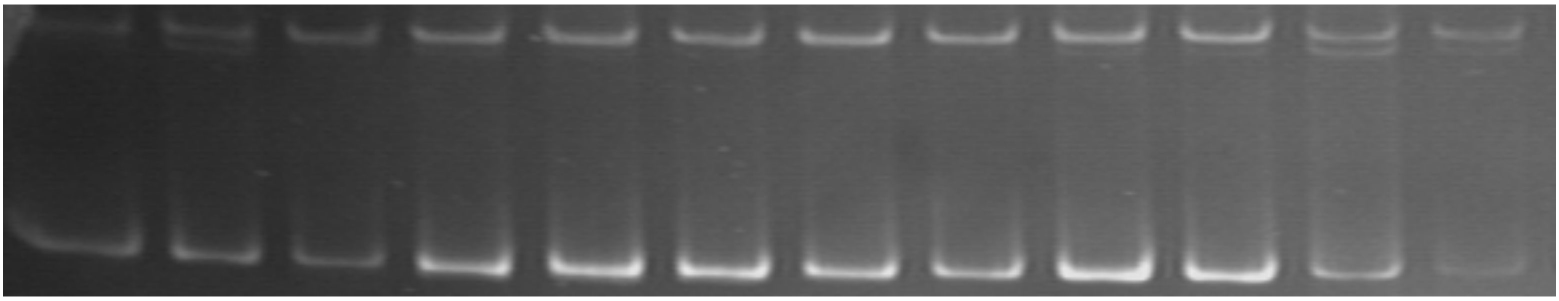

Exon 21.18

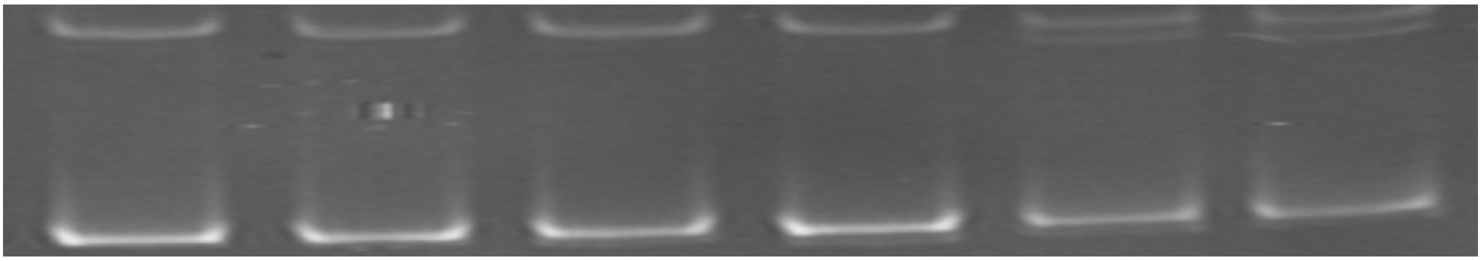

Exon 21.19

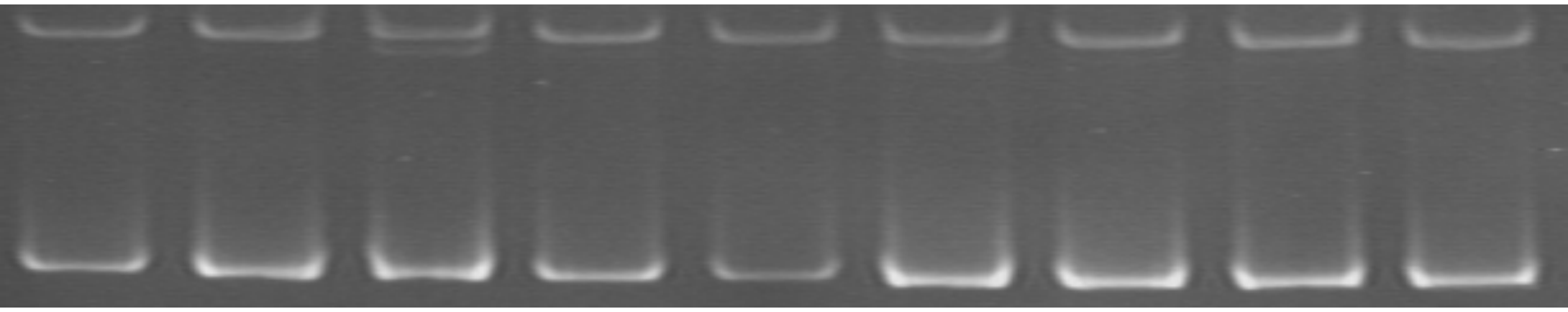

Exon 21.20

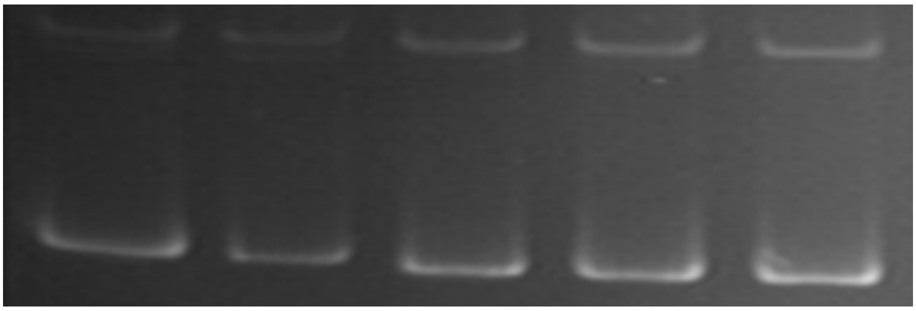

Exon 21.21

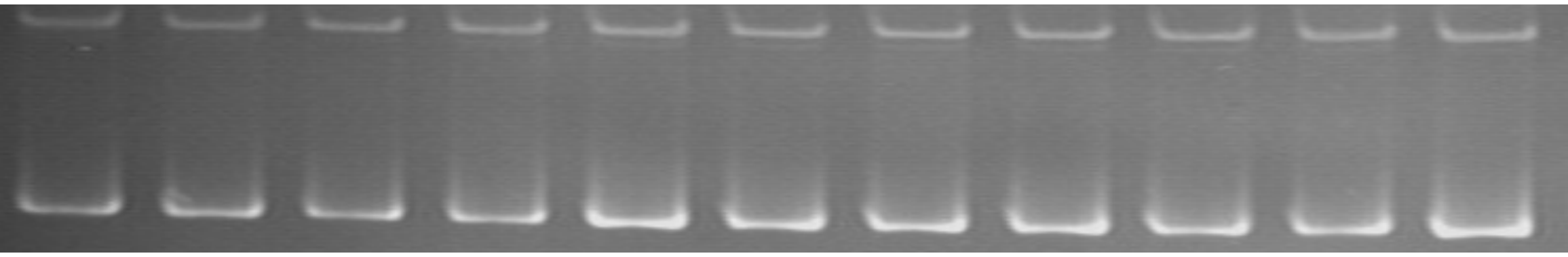

Exon 21.22

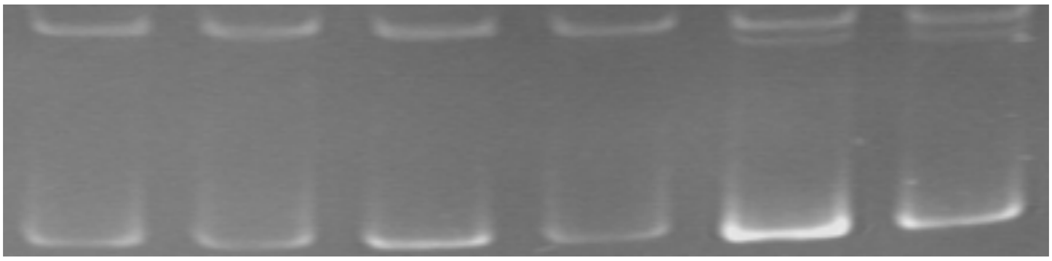

Exon 21.23

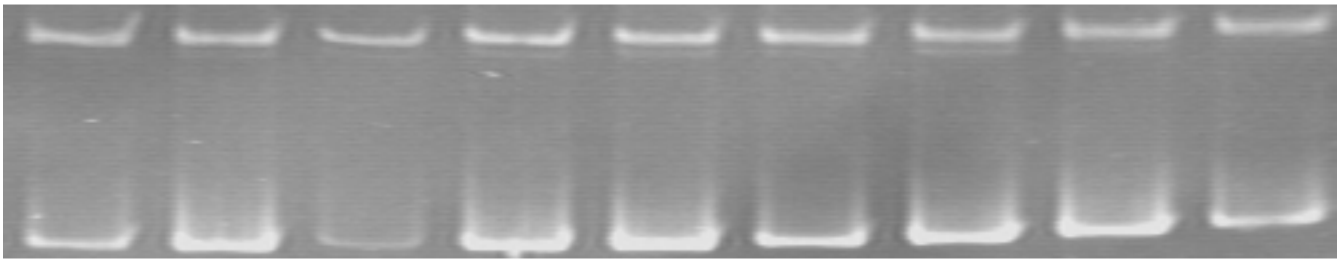

Exon 21.24

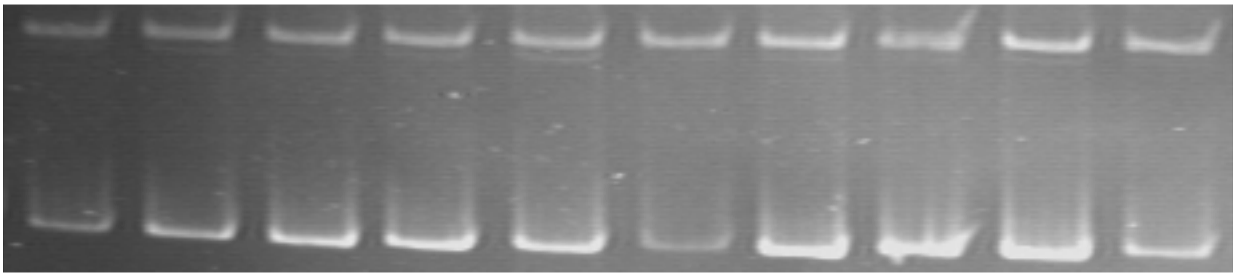

Exon 21.25

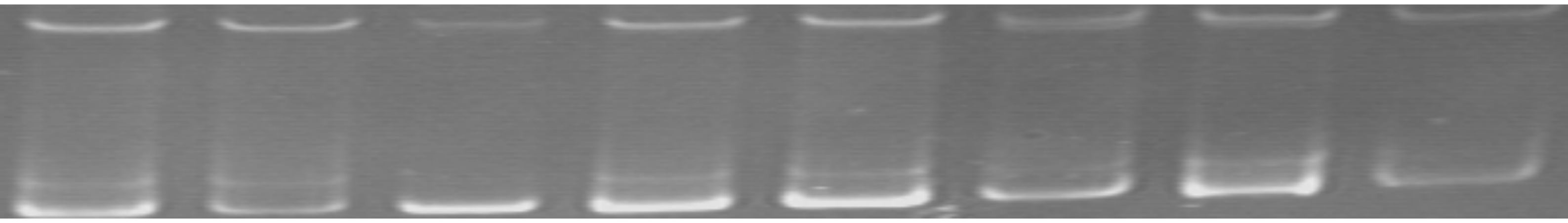

Exon 21.26

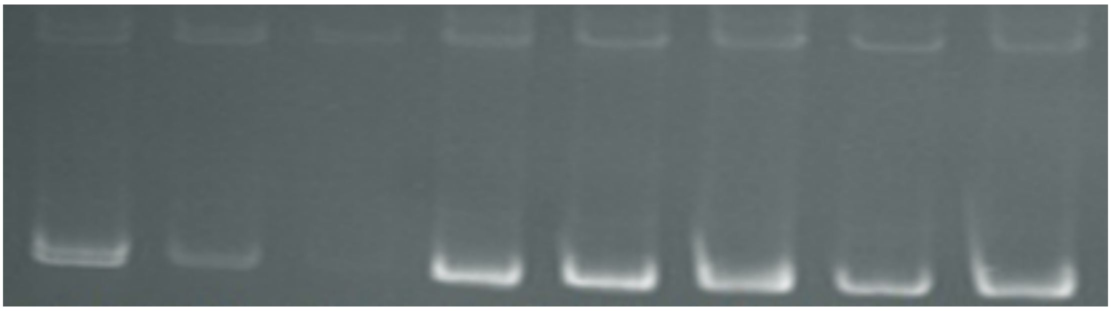

Exon 22

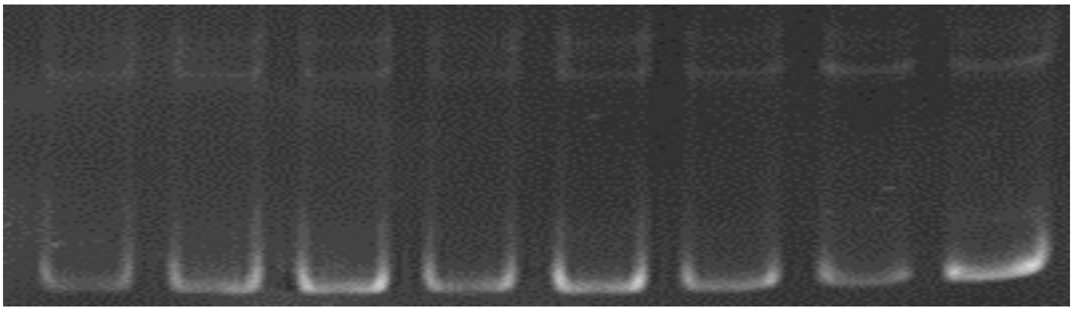

Exon 22.4

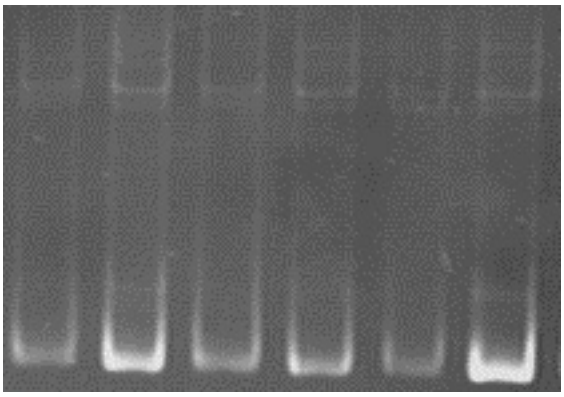

Exon 22.5

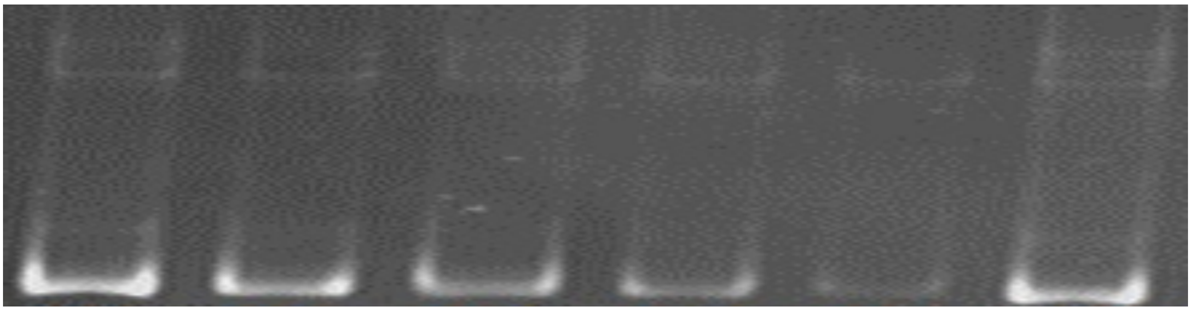

Exon 22.6

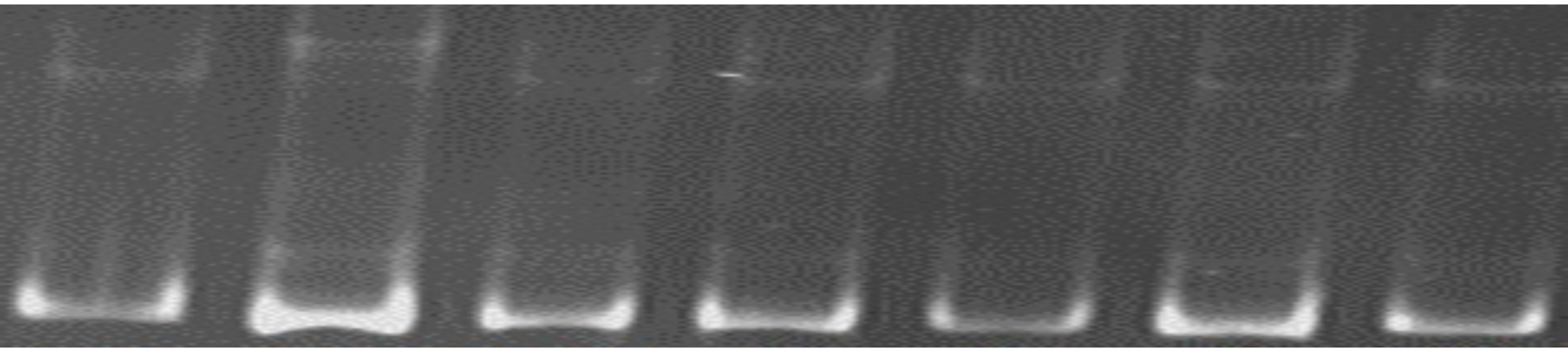

Exon 22.2

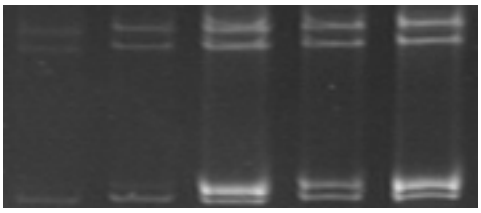

Exon 22.1

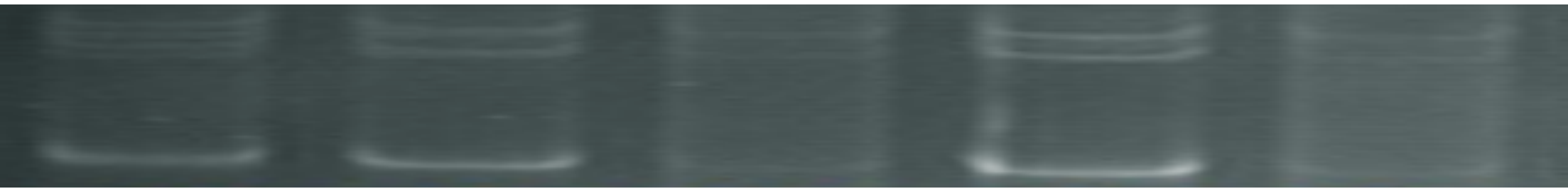

Exon 22.3

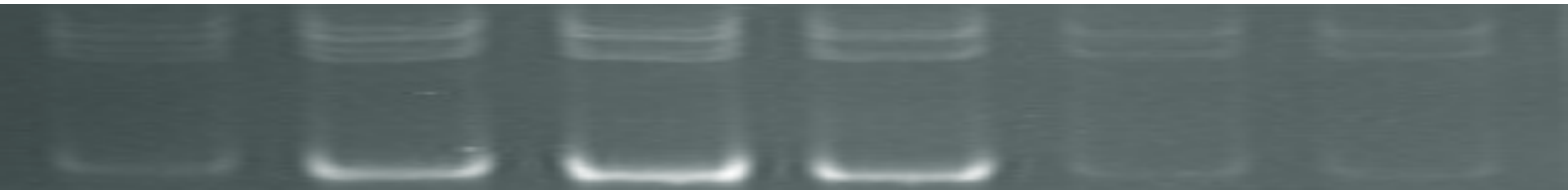

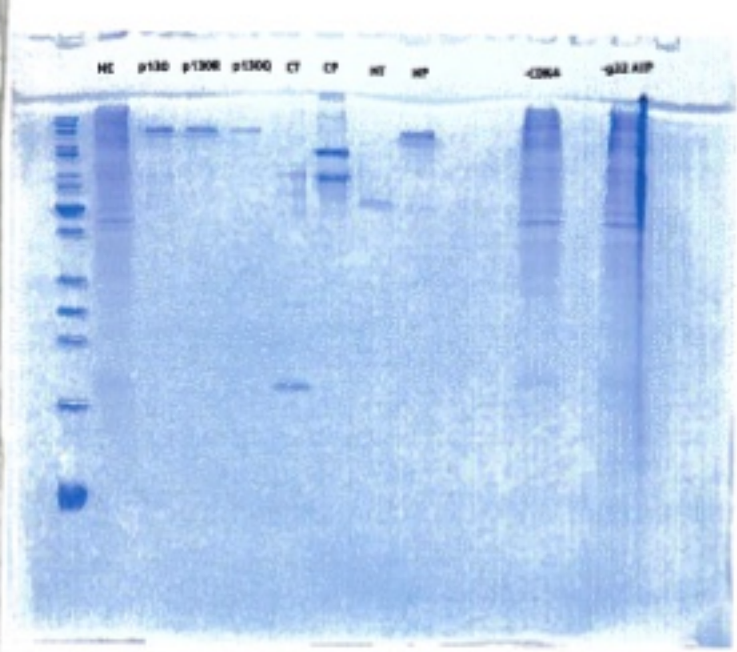
NE
p130
p130R
p130Q
CT
CP
NT
NP
-CDK4
-g32 ATP

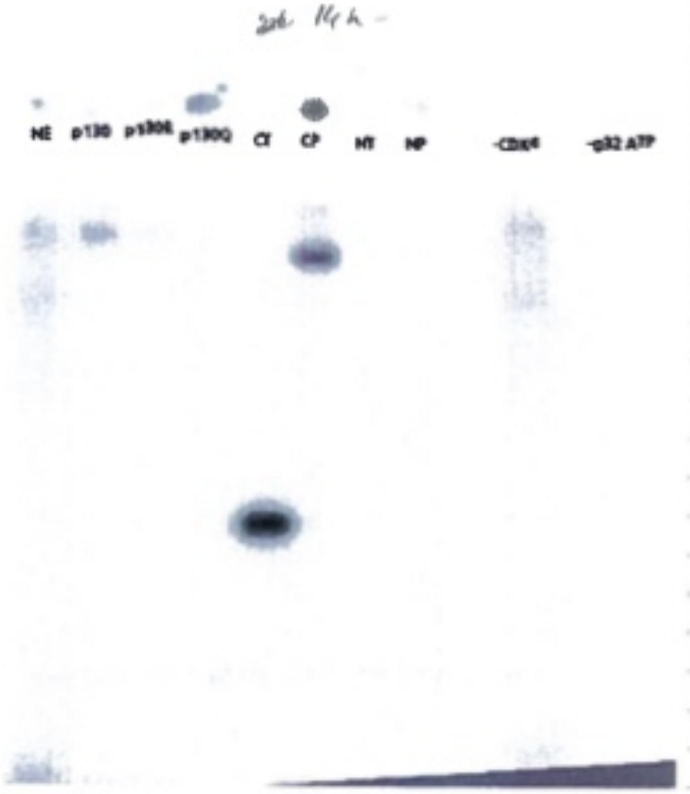
NE
p130
p130R
p130Q
CT
CP
NT
NP
-CDK4
-g32 ATP
