# Supplementary material for: Mutations in the acetylation hotspots of Rbl2 are associated with increased risk of breast cancer
Source: PLoS One. 2022 Apr 6;17(4):e0266196. doi: 10.1371/journal.pone.0266196 (PMC8985964; doi:10.1371/journal.pone.0266196)
Supplement: Attachment — Submitted filename: Reponse to reveiwers PloS 2022.docx [file pone.0266196.s003.docx]

**COMSATS University Islamabad**

Park Road, Tarlai Kalan,45550 Islamabad;

Phone: +92-51-9247000-9247002

**Department of Biosciences**

*Feb 9, 2022*

Academic Editor

Subject: **Response to reviewers of the manuscript [PONE-D-15-20345]**

Dear Editor,

The revised manuscript [PONE-D-22-02667] after making necessary changes in the light of comments given by academic editor and reviewer’s is being submitted. The academic editor and worthy reviewers have highlighted valuable points that highlight their in-depth knowledge and understanding of the subject. Both the reviewers have conceded the merit of the manuscript for publication. We are thankful for their kind comments. Following changes have been made to meet the suggestions of the worthy reviewers.

**Journal requirements:**

1. “*Please ensure that your manuscript meets PLOS ONE's style requirements, including those for file naming. The PLOS ONE style templates can be found at* [*http://www.plosone.org/attachments/PLOSOne_formatting_sample_main_body.pdf*](http://www.plosone.org/attachments/PLOSOne_formatting_sample_main_body.pdf) *and* [*http://www.plosone.org/attachments/PLOSOne_formatting_sample_title_authors_affiliations.pdf*](http://www.plosone.org/attachments/PLOSOne_formatting_sample_title_authors_affiliations.pdf)*”*

**Response:** Manuscript now meets the journal formatting requirements and all the necessary changes has been incorporated.

2. *“PLOS ONE now requires that authors provide the original uncropped and unadjusted images underlying all blot or gel results reported in a submission’s figures or Supporting Information files”*

**Response:** The raw gel figs have been included in Supporting 2.

**Rebuttal reviewer #1:**

Worthy reviewer-1 has not demanded any additional experiments to decide the merit of this manuscript. We are thankful for his/her kind opinion that manuscript is “worthy of publication”. A point wise rebuttal to all the comments are as follows.

1. *“There are many instances where acronyms are not defined upon first use. For example, what is SSCP- this should defined this when used first in the abstract. The same is true later when they first describe MDS and RMSD experiments.”*

**Response:** The point is well taken and suggestion has been incorporated in the revised manuscript.

2. *“In the very beginning of the results the authors should more clearly state the source of the material used for SSCP analysis. In the methods, it says fresh tissue was collected with adjacent normal and blood. It is not clear from the manuscript where the mutations are arising and if they are in fact more prevalent in diseases. This should be made more clear. Of note, I am not an expert on judging their frequency in tumor vs normal tissues, and these data should be reviewed by someone who is..”*

**Response:** As suggested by the worthy reviewer it has been explicitly mentioned in the beginning of results section (page 8) that

*“Genomic DNA from freshly obtained biopsies (n =200) along with their adjacent normal control tissues (ANCT) and blood samples were used to perform SSCP analysis.”*

Moreover, it has also been clarified on the same page that

“These mutations were more prevalent in tumor tissues compared to ANCT controls as has been already highlighted in table 1”

3. *“Results say direct sequencing was done on suspected individuals. What does this mean? That a potential mutation was identified by SSCP, which was then validated by direct sequencing?”*

**Response:** This has been clarified in the updated version of the manuscript. The suspected individuals were the patients that shown mobility shift in SSCP analysis. DNA from such cases were sent for sequencing.

4. “The authors say that “45 mutations were detected in these exons out of which 28 mutations were in exon-21 and 17 in exon-22.” How many samples were analyzed to identify these 45 mutations?”

**Response:** This has also been clarified in the updated version, now it reads on page 8 that

“From 200 biopsy tissues screened for genetic mutations, total 45 mutations were detected out of which 28 mutations were in exon-21 and 17 in exon-22.“

5. “The cell cycle analysis shown in table 2 is very interesting. Could these flow cytometry plots be shown?”

**Response:** The flow cytometric plots has been added as figure 6 in the updated manuscript.

6. “Are p130(Q) cells arrested in G2? Could the authors elaborate on why this might be the case?”

**Response:** The observation that noticeable population of ‘K1083Q” mutants remained present in G2/M phase is highlighting the significance of acetylation at this particular residue and this is also thrilling for all of us. In the updated version On page 10 and in discussion page 14, we discussed the possible consequences of this mutation that may lead to perturbed cell cycle. On page 14 it reads as

“Since K1083Q mutants mimic acetylated form (in reference to steric charge) of Rbl2, they might imbalance phosphorylation status of Rbl2 that resulted in the abolishment of G1/S checkpoint. Hence, they remained predominantly in G2/M phase of cell cycle.”

7. “The source of the structure of RBL2-cyclin D show in Fig 6 should eb stated in the results section and not only in the methods. As it reads, in the results, it was unclear if that was modeling of a previous structure or the authors own structural data. This should be made clearer.”

**Response:** In revised version it is Fig 7In order to clarify the points following para has been on page 11.

**“**To study kinetics of interaction between residue K1083 and Cyc-D1/CDK4T, the molecular docking and simulation studies were performed. The structure of Cyc-D1/CDK4 complex was retrieved from protein data bank (entry code: 2W96), whereas the C-terminal domain of wild-type Rbl2 and its mutated forms i.e. K1083R, K1083Q, and K1083D were modelled through ab initio modelling server “Quark”.

8. “The source of the material used in the final figures should be shown, to demonstrate how pure that protein was, and it should be made clear in the results what the source of that protein is, and more clearly stated how those experiments are done, analyzed and interpreted. These experiments are also outside of my general expertise and should be reviewed by someone more capable of judging those data.”

**Response:** The query has been resolved by mentioning the sources of *in silico* models used for molecular dynamic studies. It is to bring into notice that the said investigations don’t involve wet lab experiments, where highly purified proteins are required to perform interaction studies.

**Rebuttal reviewer 2:**

Worthy reviewer-2 has also appreciated the data presented in the manuscript and has positively commented on the scope of data to elucidate cell cycle kinetics in further details. We are really thankful for his/her kind words. However, the reviewer has suggested to perform additional experiment to confirm that changes in C-terminal Rbl2 sequences may hinder its association with cyclinD1-CDK4 complex. This will support the *in-silico* observations that C-terminus and K1083 in particular is involved in this interaction.

**Response:** We’re glad to be able to show that without C-terminus an interaction with cyclinD1-CDK4 is not possible. The N-terminus without an intact C-terminus is unable to undergo phosphorylation in the presence of cyclinD1-CDK4 complex. These *in vitro* kinase assay supports our *in-silico* observations that C-terminus is involved in this interaction. Moreover, a mutation at K1083 also weakens this association as is evident from the week phosphorylation status of these protein. The results have been included in the updated version of manuscript and are presented in figure 9.

We are again thankful for valuable feedback of academic editor and both reviewers.

Submitted for onward processing

Sincerely,

Muhammad Saeed, PhD

Associate professor
