# Supplementary material for: Mutations in the acetylation hotspots of Rbl2 are associated with increased risk of breast cancer
Source: PLoS One. 2022 Apr 6;17(4):e0266196. doi: 10.1371/journal.pone.0266196 (PMC8985964; doi:10.1371/journal.pone.0266196)
Supplement: Attachment — Submitted filename: Reponse to reveiwers-2.docx [file pone.0266196.s004.docx]

**COMSATS University Islamabad**

Park Road, Tarlai Kalan,45550 Islamabad;

Phone: +92-51-9247000-9247002

**Department of Biosciences**

*March 10, 2022*

Academic Editor

Subject: **Response to Academic Editor of the manuscript [PONE-D-15-20345]**

Dear Editor,

The revised manuscript [PONE-D-22-02667] was submitted to your office after making necessary changes in the light of comments given by academic editor and reviewer’s. Although we have addressed all the points highlighted by worthy reviewers, somehow, we missed to respond this final comment. We sincerely apology for that.

**Comment:**

1. *“-In addition, evaluating the expression of RBL2 mutant in relevant acetylation sites and their effect on cell cycle would be another important experiment to include.”*

**Response:** We apricate these comments to explore the effect of mutant Rbl2 expression at various acetylation sites on cell cycle. Current study identified 45 mutations in total and out of these 45 mutations 10 missense mutations were observed in/around NLS (Fig 2A). Three acetylatable residues K1072, K1083 and K1115 were specifically scanned for mutations. Only K1083 was found altered into an arginine (R), Whereas K1072 and K1115 remained conserved although a silent mutation at K1072 was observed in few primary tumors (Fig 2B). The Kaplan-Meir analysis presented in Fig 4 & 5 reflects that only mutation at K1083 resulted in poor survival outcome of patients.

Since we observed acetylatable residue K0183 only as a clinically meaningful factor for survival analysis, we performed *in vitro* mutagenesis experiments to explore its impact on cell cycle (Fig 6 and Table 2).

In this context, we believe that worthy reviewer’s comment has already been addressed. Moreover, cell cycle analysis on K1072 and K1115, although is interesting academically, but might not be clinically significant and is beyond the scope of current manuscript

Further, *In silico* analysis revealed that K1072 & K1115 don’t interact with Cyclin/CDK4 complex, again highlighting that these residue might not have clinical impact. In this revised version we have included the table (Table 3) of interaction that further support that why cell cycle analysis of K1083 was performed only.

We are again thankful for valuable feedback of academic editor and both reviewers.

Submitted for onward processing

Sincerely,

Muhammad Saeed, PhD

Associate professor
